# Development of a Protocol Using the Delphi Method for the ad interim Supply of Hormonal Contraceptives in Swiss Pharmacies

**DOI:** 10.3390/pharmacy10060168

**Published:** 2022-12-02

**Authors:** Tamara Yous, Esther Spinatsch, Samuel Allemann, Monika Lutters

**Affiliations:** 1 Department of Medical Sciences, Private Universität of the Principality of Liechtenstein, 9495 Triesen, Liechtenstein; 2 Pharmaceutical Care Research Group, Department of Pharmaceutical Sciences, University of Basel, 4056 Basel, Switzerland; 3 Hospital Pharmacy, Cantonal Hospital of Aarau, 5001 Aarau, Switzerland

**Keywords:** pharmaceutical service, pharmacist prescribing, community pharmacy, birth control, eligibility for contraceptives, contraception, behind the counter, patient safety, women’s health

## Abstract

(1) **Background:** Pharmacists are often challenged with situations where women are already on hormonal contraceptives (HC) but have no valid prescription. By Swiss law, pharmacists are allowed to supply prescription-only drugs in exceptional situations without a physician’s prescription. Because eligibility for HC can change, women at risk for complications, such as serious side effects, need to be identified. We aimed to develop a protocol to assist pharmacists in clarifying and documenting eligibility for HC. (2) **Methods**: We conducted a survey using the Delphi method to identify relevant clarifications and develop a protocol for pharmacists. Proposed material was created based on the literature and existing toolkits/protocols aimed at verifying eligibility for HC. A multidisciplinary expert panel, consisting of gynecologists and pharmacists, reviewed the proposed material and provided anonymized feedback over two survey cycles. (3) **Results:** This Delphi survey revealed items essential to the clarification of eligibility for HC in pharmacies for women who are already using it. This resulted in a protocol that maps “best practices” regarding these ad interim supplies of HC given without a prescription in Switzerland. (4) **Conclusions**: This survey, made using the Delphi method, allowed us to create a protocol for pharmacists that aims to verify and document eligibility for HC in Switzerland, where HC is frequently supplied without a prescription.

## 1. Introduction

Accessibility to hormonal contraceptives (HC) varies widely around the world and ranges from prescription-only status to behind-the-counter strategies to over-the-counter availability. Even among European countries, accessibility differs significantly. In the European Contraception Policy Atlas, published by the EPF (European Parliamentary Forum for Sexual and Reproductive Rights), countries are stratified (with ranking points given in percent and by traffic light colors) according to their access to contraceptive supplies, family planning counseling, and online information [1]. Belgium, France, and the United Kingdom topped this ranking list (91.1%, green light); in contrast, Poland reached last place (33.5%, red light). The ranking for Switzerland was only mediocre (58.3%, yellow light) and the experts of the EPF recommended that self-administered HC be made available without prescription to reduce access barriers [2]. Overall, the analysis revealed an uneven picture across Europe and the authors concluded that access to modern, effective, and affordable contraception remains a challenge in Europe.

In 2019, a new law was introduced by the Swiss government with the aim of simplifying access to certain medicinal products subject to prescription [3]. Under this new law, pharmacists may directly supply medicinal products subject to prescription under certain conditions. To our knowledge, the decision to allow the supply of HC without prescription is still pending. Our previous research showed that pharmacists are regularly challenged with situations in which no valid prescription for HC is available and they must decide whether or not to dispense without a prescription [4]. About 97% (*n* = 320/331) of participating pharmacists answered that they supply HC without a prescription. Others have noted the high prevalence of pharmacists dispensing HC without a prescription to women who are already using it [5]. The authors of this mixed-method approach from Belgium concluded “that this practice [supply of HC from pharmacists without a prescription] anticipates what a large proportion of health care providers suggested or could agree with: extending a prescription to the pharmacist.”

Due to easy access and long opening hours, pharmacies are already a frequent contact point for women who urgently need HC. By law, pharmacists in Switzerland are authorized to supply prescription-only drugs (including HC) without a prescription in exceptional situations [3]. However, documentation is required and dispensing prescription-only drugs without a prescription is a responsibility that lies with the pharmacist. Although most women can safely take HC, and the advantages outweigh the possible risks in most cases, eligibility needs to be checked regularly because contraindications may change over time [6]. Fortunately, serious complications like venous thromboembolism or brain and myocardial infarction are rare in women of reproductive age [7,8]. The Medical Eligibility Criteria (MEC) provides guidance about which women can use contraceptive methods safely, e.g., UKMEC [9]. Depending on the situation, checking eligibility before supplying HC without a prescription may be important for pharmacists since the supply is their responsibility. So far, there is no guidance regarding these situations in Switzerland. The main goal of this study was to develop a protocol that aims to verify eligibility in women who are already on HC and need an ad interim supply from pharmacists.

## 2. Materials and Methods

The Delphi method can be used to collect expert judgments and identify consensus [10,11,12]. Using this method, we developed a protocol for pharmacists in Switzerland that aims to verify eligibility in women who are already using HC but have no valid prescription. An interdisciplinary expert panel was recruited for this Delphi survey and participating experts were identified via professional networks and peer recommendations. Recruitment took place by email and the survey was conducted in German. The panel consisted of 19 experts from Switzerland (8 gynecologists and 11 pharmacists). The first group was composed of hospital and practice-based gynecologists, including gynecologists focusing on pediatrics and adolescents. For the second group, pharmacists either working in a community pharmacy, involved in university education or further training in the field of sexual health were deemed suitable for participation.

The proposed material was created based on specialist literature [6,13] and existing toolkits/protocols [9,14,15,16,17,18,19,20,21] aimed at verifying eligibility for HC. We developed a protocol for our Delphi survey similar to that of Meredith et al. to enhance pharmacist contraceptive counseling materials [22]. Experts reviewed the proposed material and gave anonymous feedback using the web-based survey tool SoSci Survey (Version 3.2.55) [23]. Requested feedback from experts focused mainly on clarifications or answer options and whether they were important in the context of supplying HC without a prescription. Layout or visual appeal were not a subject of the survey.

Furthermore, a commentary sheet with additional counseling information for pharmacists was created, as this is provided in other Supplementary Materials for pharmacies, e.g., for the supply of emergency contraception [24]. This material was reviewed for optimization but is not included in this manuscript (see Supplementary Materials).

**Figure 1:**
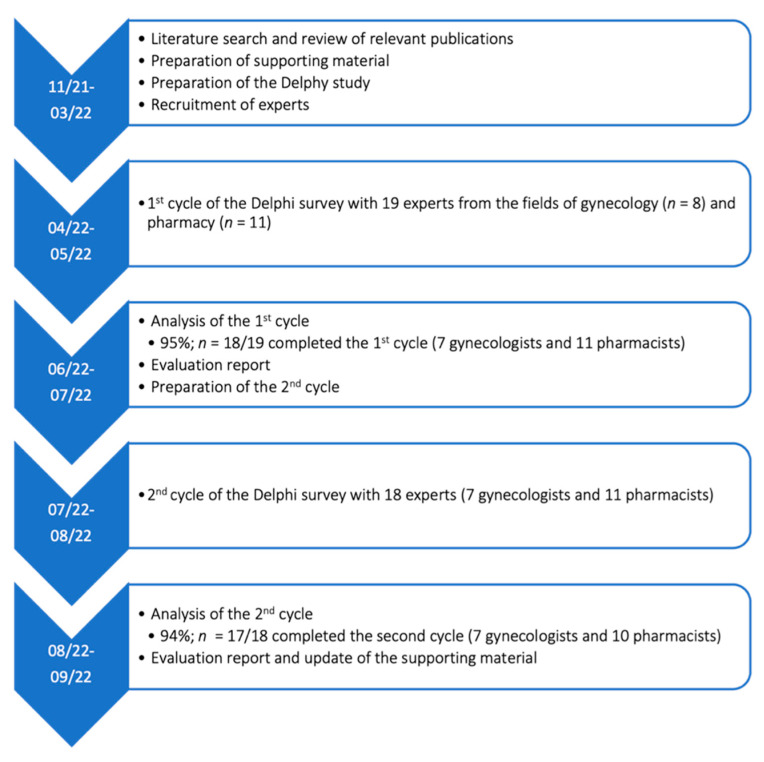
Flowchart of the Delphi process.

We conducted this Delphi survey in two cycles (Figure 1). The first cycle was carried out from April to May 2022 and the second cycle took place from July to August 2022. During the consensus-finding process, the experts determined, in both cycles, whether a certain item is important for clarification or documentation and should be included in the protocol. A 4-point Likert scale was used for the evaluation of inclusion (1 = no; 2 = rather no than yes, 3 = rather yes than no, 4 = yes; 0 = abstention). Furthermore, experts could propose new items or reformulations. A consensus was defined a priori for the first cycle based on 80% agreement for inclusion (“yes” or “rather yes than no”). To meet inclusion criteria, consensus needed to be achieved in both groups (gynecologists and pharmacists), and groups were weighted equally. Based on results and feedback received in the first cycle, relevant modifications, reformulations, and alternative proposals were subject to a vote in the second cycle. If appropriate, a tie-breaker question was inserted to obtain a clear opinion on which version is preferred (majority decision, no predefined agreement level). Borderline results (61–79%) from the first cycle were put to vote again with a brief comment on why this item was previously proposed. In the case of unclear results, arguments and comments from the second cycle were considered for the final decision. Adjusting for abstentions and dropouts in the second cycle, we aimed for 70% agreement in both groups, which was defined a priori. The feedback was analyzed quantitatively using SPSS^®^ (IBM Corp. Released 2020. IBM SPSS Statistics for Mac, Version 27.0. Armonk, NY, USA) and Microsoft^®^ Office Excel (for Mac, Version 16.50) for the overview of results and visualization. Evaluation reports with relevant voting results were sent to the experts after every survey cycle.

## 3. Results

The final protocol is divided into four sections: (I) short clarification, (II) detailed clarification, (III) information given to the woman, and (IV) decision documentation. The first section includes general clarifications that should be sought for every woman asking for HC without a prescription. In this part, pharmacists gain information about the reason why no valid prescription is available and whether HC have been prescribed by a physician in the last two years. In addition, questions about the product, if it is tolerated well, and the date and quality of the woman’s last menstruation are also included. At the end of the first section, pharmacists decide if more detailed clarification regarding eligibility for HC is needed (section II). In the third and fourth section of the protocol, information given to the woman and the pharmacist’s decisions can be documented.

### 3.1. Results from the First Cycle

During the first cycle, one gynecologist withdrew due to lack of time, leading to a participation rate of 95% (*n* = 18/19). Of the 106 items proposed for voting, 58 items met the inclusion criteria, 11 items were rejected, and 38 items did not yield clear results. An overview of relevant voting results is displayed in Table 1. Regarding documentation pertaining to why an urgent supply is needed, participants wished for another answer option: “HC are usually obtained from the physician.” The proposed time period of two years (since the last prescription was issued) was actively commented on. The participating gynecologists deemed this period to be the maximum in principle. Therefore, we incorporated a vote in the second cycle about the appropriateness of this time period. At first, separate lists for potential contraindications and risk factors were presented, for which experts left comments such as “too extensive” or “like interrogation.” Therefore, the voting results, summarized in Table 2, do not include a decision for inclusion; instead, a shorter alternative was proposed in the second cycle. Clarification about smoking, and especially the cut-off of 15 cigarettes per day, was discussed with much controversy, and a more pragmatic option was proposed afterwards. Two gynecologists stated that no combined HC (CHC) should be given if a woman is over 35 years old, regardless of the number of cigarettes smoked per day. Furthermore, pharmacists wished to use layman’s terms instead of professional jargon. This feedback was received particularly for the section on detailed clarification (section II).

### 3.2. Results from the Second Cycle

Out of the 18 experts from the first cycle, 17 participated in the second cycle (94%; *n* = 17/18). Although, one survey was not fully completed, it was included for analysis and had no major impact on results. The second cycle contained mainly revotes for items with unclear results from the previous cycle, as well as votes about reformulations and alternative proposals. A total of 67 items were presented. For some topics, e.g., clarification regarding smoking, more than one option was presented for voting (reformulations, alternative proposals, or tie-breaker questions). In total, 27 items achieved agreement and were incorporated into the protocol. Most relevant results are displayed in Table 3. Due to a tight voting result, the question “Do you tolerate the product well?” was presented again in the second cycle and arguments from experts were included in the decision-making process. A typical physician’s comment was: “If the woman did not tolerate the preparation well, she would probably not want to continue taking it.” Pharmacists clearly voted for the incorporation of this clarification: “This is a fundamental question in pharmacotherapy”; “Clarification about tolerance should be addressed”; “If there are issues, the therapy should be optimized.” Furthermore, this question was seen as a “door opener” for the pharmaceutical conversation, “giving the chance to address also further issues like the loss of libido or mood changes/depression.” Therefore, this clarification was incorporated into the protocol, although consensus was not achieved. What is noteworthy is that more than half of the participating gynecologists voted for the inclusion. The simplified clarification about smoking (answer options: daily or occasionally instead of a cut-off with 15 cigarettes per day) was preferred by both groups in the tie-breaker question (gynecologists: 100%; pharmacists: 60%). However, the alternative proposal, which already included a clarification about smoking, was accepted with higher agreement. Details regarding this alternative proposal to clarify contraindications and risk factors are summarized in Table 4. The majority in both groups voted for the incorporation of the alternative proposal in the tie-breaker question (gynecologists: 67%; pharmacists 80%). The final protocol can be found in the supplement accompanying this article.

## 4. Discussion

The goal of this study was to develop a pharmacist’s protocol for the ad interim supply of HC, aiming to verify eligibility in women who are already using it but have no valid prescription. Use of the Delphi method allowed us to identify relevant clarifications by collecting feedback from experts in gynecology and pharmacy. The survey focused on a protocol assisting pharmacists in verifying and documenting eligibility for HC using a few questions, as well as identifying women at risk, e.g., for complications such as serious side effects, who might be referred to a physician. Over two survey cycles, the proposed material was reduced to items essential to the common practice of supplying ad interim HC in pharmacies. The protocol was structured similarly to the protocol for emergency contraception that is already frequently used by pharmacists in Switzerland [25].

In most cases, the voting results clearly showed whether a particular item should be included into the protocol or not. One topic for which we received additional feedback, and which did not yield a clear result in the first cycle, was the clarification about smoking. In accordance with the UKMEC, we initially proposed to ask smokers whether they smoke more or less than 15 cigarettes per day. Some participants, however, have recommended a simplification, which resulted in acceptance in the second cycle. By contrast, two pharmacists reported back that the clarification should be carried out in line with the existing guideline with the initially proposed cut-off of 15 cigarettes. We think that the simplified version is sufficient for the special situation of ad interim supply in pharmacies because pharmacists in Switzerland are currently not authorized to prescribe HC. Furthermore, votes on contraindications and risk factors showed inconsistent results after the first cycle. In the beginning, separate clarifications for possible contraindications and risk factors were proposed but results showed that experts aimed for a more pragmatic option. Therefore, an alternative proposal was presented in the second cycle, based on an information leaflet for women created by the Swiss Society for Gynecology and Obstetrics (SGGG) [21]. This leaflet was originally designed for women using CHC and included the most relevant contraindications and risk factors. This leaflet also mentioned two conditions, “valvular hearth disease” and “prolonged bed rest,” which did not achieve consensus in our survey. This might be explained by the fact that valvular heart diseases are rare conditions in young women and that these women probably see their physicians regularly and should already be aware of potential risks. Customers with prolonged bed rest will most probably not visit a pharmacy, and in cases of planned surgeries, this topic should be addressed by other involved health care professionals (HCPs), e.g., surgeons or anesthetists. Interestingly, pharmacists voted for the inclusion of this item, while gynecologists saw this as irrelevant to this situation. This result underlines the willingness of participating pharmacists to seek in-depth clarifications regarding birth control services. Another difference to note was that gynecologists preferred a detailed clarification with a check for eligibility every time HC/CHC are dispensed without a prescription. In some situations, this seems excessive, e.g., when the prescription had expired a few days earlier. Therefore, pharmacists should decide on a case-by-case basis.

Regarding the appropriateness of the time period of two years (since the last prescription was issued), it is worth mentioning that long-term prescriptions for HC are often already issued for one to two years or for a corresponding number of packages. The validity of prescriptions is regulated by the Canton, and therefore different validity periods exist across Switzerland. For example, in Zurich, St. Gallen, and Lucerne, long-term prescriptions are already valid for two years [26]. Therefore, further restriction would not be effective and the majority in both groups voted that the time period of two years seemed justified.

A general clarification about allergies and intolerances did not result in consensus. To create a practical and concise protocol, this clarification was not included since it is also not specifically related to HC. Normally, the same product will be given by the pharmacist, making this question redundant. However, we considered this clarification reasonable when switching to another product, e.g., due to supply shortages. Therefore, we suggested that this question should be asked only if needed. Furthermore, it was not surprising that pharmacists preferred layman’s terms, which can be used directly with their clients. In addition, more pharmacists voted for the inclusion of a clarification as to whether emergency contraception has been taken in the last three months. This question might be useful, especially before renewing a prescription for HC, to ensure that the birth control method is still practicable and “the right one.” Since Swiss pharmacists often provide an ad interim supply and are currently not authorized to issue follow-up prescriptions, gynecologists considered this clarification less necessary for this particular situation. Furthermore, women should receive all information needed during counseling on emergency contraception.

When a contraindication or important risk factor seems present and has not already been discussed with a physician, a referral is indicated. It is important that pharmacists decide on a case-by-case basis and make a balanced decision. The decision to supply HC should be based on the individual situation and is the responsibility of the pharmacist. For example, the UKMEC offers guidance to providers of contraception regarding the question of who can use contraceptive methods safely and offers more information about risk-benefit ratios [9]. Potential health-related risks need to be considered, but the risk and consequences of a possible unintended pregnancy should also be taken into account. Importantly, pharmacists are always allowed to ask more, and it is essential that they adapt their pharmaceutical conversation to each situation. Due to many different situations in pharmacies, there is no single correct way to clarify eligibility before dispensing HC ad interim. It is therefore difficult to propose a standard protocol. Nevertheless, having an expert panel consisting of both gynecologists and pharmacists allowed us to identify “best practices” for counseling in pharmacies. This protocol applies to the ad interim supply of HC and assists pharmacists in their daily practice; it may also contribute to patient safety.

### 4.1. Outlook

This protocol can serve as a template for professional bodies and policy makers regarding further discussions about the extended involvement of pharmacists in birth control services. Access to contraception is determined by multiple factors, such as affordability, availability, and access to information. Women can face different barriers and extended access to HC is a hot topic. Extended access to HC has already been introduced in different countries and has been shown to be feasible and safe when provided by appropriately trained HCPs, such as pharmacists [27,28,29,30,31]. While in the United States of America prescription status remains unchanged, many states allow pharmacists to initiate HC or renew prescriptions [27]. Authorizing pharmacists to prescribe HC would also be a major change in the Swiss health care system. However, based on the recommendations of the EPF (to reduce access barriers and make HC available without prescriptions) [2], together with the new Swiss law [3], extended access to HC could be a new strategy in Switzerland and should be further discussed. Importantly, we found pharmacists participating in our survey to be motivated and willing to train for this new service (90%; *n* = 299/331) [4]. In addition, our previous survey among physicians practicing in Switzerland showed that a combined access model (initial prescription by physicians and follow-up prescriptions from pharmacists) also found wide acceptance (70%; *n* = 103/147) [32].

### 4.2. Strengths and Limitations

Our study has various strengths. This is the first instance of identifying the most relevant clarifications that should be made before pharmacists in Switzerland dispense HC without a prescription. For this purpose, we used the Delphi method and recruited expert groups, consisting of gynecologists and pharmacists practicing in Switzerland. Furthermore, the proposed clarifications are based on the relevant literature and existing tools aimed at verifying eligibility for HC; these clarifications are therefore evidence-based. Feedback of the experts allowed to adjust or reformulate certain topics to local conditions (e.g., wording, legal regulations, and current practice) and reflect “best practices” for the current practice of the ad interim supply of HC in Swiss pharmacies. Our study, using the Delphi method, also has some limitations, like the potential for bias in the selection of experts and the limited time of experts participating in multiple cycles. To respect the experts’ time, the requested feedback was kept as short as possible. Sometimes, certain clarifications and feedback required simplification for this project and not all ideas could be utilized. Furthermore, the protocol was developed in German. In addition, considerations regarding layout were not part of this research; since the supply is usually digitally registered with a pharmacy software, further layout optimization is secondary.

## 5. Conclusions

Use of the Delphi method identified relevant clarifications that should be made before pharmacists dispense HC to women who are already using it but have no valid prescription. This survey revealed “best practices” for this type of counseling in pharmacies and allowed for the creation of a protocol to verify eligibility. This protocol can be used for the frequently practiced ad interim supply of HC in Swiss pharmacies. In addition, it can serve as a basis for further discussions regarding access to HC from pharmacists in Switzerland.

## Figures and Tables

**Table 1:** Relevant Voting Results from the First Cycle.

Clarification	Agreement (%)		Inclusion ^◊^
	Gynecologists	Pharmacists	
What is the reason for the urgent supply of HC without a Rx?	100	100	Yes
*Answer options:*			
(a) Rx expired on...	100	100	Yes
(b) Prescription not available/present	100	90	Yes
(c) Foreign Rx	100	82	Yes
(d) Other	67	100	Yes ^#^
Have HC been prescribed by a physician within the last 2 years?	100	100	Yes
Which product do you take, or do you use?	86	100	Yes
When did you last take, or use the product?	71	82	*
Do you tolerate the product well?	71	91	*
When was the last gynecological check-up?	86	100	Yes
When did the last menstrual bleeding start?	100	91	Yes
How was the pattern of the last bleeding?	86	64	^&^
Have you been taking EC in the last 3 months?	57	73	No
Have you taken a pregnancy test after using EC?	57	56	No
Can a pregnancy be excluded with a high degree of probability?	100	90	Yes
Does the detailed clarification make sense?	83	90	Yes
*Examples, when a more detailed clarification should be done:*			
(a) For every expired Rx	50	36	No
(b) Every time HC are dispensed without Rx	71	30	^&^
(c) Missing evidence for previous Rx of HC	100	64	*
(d) Foreign Rx	83	64	*
(e) Gynecological check-up of HC > 1 year ago	67	46	^&^
(f) In case of uncertainty regarding eligibility for HC, e.g., woman reports a possible CI	100	100	Yes
Body Mass Index	71	82	*^,&^
Do you smoke?	86	100	Yes
cut-off: 15 cigarettes	67	70	^&^
Have you given birth within the last 6 weeks?	86	100	Yes
Are you breastfeeding?	86	100	Yes
What other drugs are you taking or using?	86	100	Yes
*If drugs are taken*: Drug evaluation by pharmacists (CI/IA)	83	91	Yes
Do you have any known allergies or intolerances?	71	100	*
Have you been diagnosed with an increased risk of thrombosis?	86	100	^&^

Participants: *n* = 18; CI = Contraindication(s); EC = Emergency contraceptive(s); HC = Hormonal contraceptive(s); IA = Interactions(s); Rx = Prescription; ^◊^ = Consensus defined a priori based on 80% agreement in both groups; # = Inclusion due to documentation purposes; * = Revote in the second cycle; ^&^ = Reformulation/alternative proposal presented in the second cycle.

**Table 2:** Voting Results Regarding Possible Risk Factors and Contraindications from the First Cycle (without Decision for Inclusion).

Clarification	Agreement (%)	
	Gynecologists	Pharmacists
Which of the following diseases/situations are known to you?	86	91
*List of contraindications for certain HC:*		
(a) Diabetes mellitus with nephro-/retino-/neuropathy	86	70
(b) Hypertension (>140/90 mmHg)	86	82
(c) Cardiovascular disease, e.g., myocardial infarction	86	82
(d) Prolonged immobilization, e.g., accident/surgery	71	73
(e) Liver dysfunction or acute hepatitis	71	73
(f) Lupus with vascular involvement	71	73
(g) Breast carcinoma, hormone-dependent carcinomas	71	82
(h) Migraine with aura	86	73
(i) Multiple sclerosis with immobility	71	73
(j) Vaginal bleeding not clarified	86	82
(k) Severe hypercholesterolemia, hypertriglyceridemia	86	82
(l) Status after venous thrombo-/pulmonary embolism	86	82
(m) Thrombophilia	86	82
(n) Cerebrovascular event	86	82
(o) None of the listed diseases/situations known	71	80
Which of the following risk factors are known to you?	71	60
*List of risk factors for certain HC:*		
(a) Diabetes mellitus without nephro-/retino-/neuropathy	71	40
(b) Dyslipidemia	71	40
(c) Inflammatory bowel disease	71	50
(d) Gallbladder disease	57	50
(e) Hypertension, well controlled	71	60
(f) Lupus without vascular involvement	71	40
(g) Migraine without aura	71	40
(h) Renal failure	71	50
(i) Organ transplantation	57	50
(j) Positive 1st degree family history for venous thromboembolism/pulmonary embolism.	71	60
(k) Other diseases (see UKMEC): ....................	71	50
(l) None of the listed risk factors known	71	60

Participants: *n* = 18; HC = Hormonal contraceptives; UKMEC = U.K. Medical Eligibility Criteria.

**Table 3:** Relevant Results About Revotes and Reformulations (Second Cycle).

Clarification	Agreement (%)		Inclusion ^◊^
	Gynecologists	Pharmacists	
*Additional voting about the accuracy of the time period since the last prescription was issued by a physician:* “within the last 2 years”	72	100	Yes
When did you last take, or use the product?	100	90	Yes
Do you tolerate the product well?	57	90	Yes ^#,ç^
Was the last period normal/as usual?	100	100	Yes
*Examples, when a more detailed clarification should be done:*			
(a) Every time CHC are dispensed without Rx	71	12	No
(b) Missing evidence for previous Rx of HC	100	90	Yes
(c) Foreign Rx	86	30	No
(d) Gynecological check-up/re-evaluation of HC > 1 year ago	72	50	No
Do you have any known allergies or intolerances?	57	89	No
Have you or a 1st degree relative had a blood clot in a blood vessel?	100	90	Yes

Participants: *n* = 17; ^◊^ = Consensus defined a priori based on 70% agreement in both groups; CHC = Combined hormonal contraceptive(s); HC = Hormonal contraceptive(s); Rx = Prescription; # = Inclusion due to documentation purposes; ç = Decision due to expert’s argument/feedback.

**Table 4:** Alternative Proposal Regarding Possible Contraindications and Risk Factors (Second Cycle).

Clarification	Agreement (%)		Inclusion ^◊^
	Gynecologists	Pharmacists	
Does one or more of the following situations apply to you?	100	90	Yes ^&^
(a) Age > 35 year	100	90	Yes
(b) Smoking	100	90	Yes
(c) History of blood clots, including 1st first degree relative	100	80	Yes
(d) Overweight	100	90	Yes
(e) (High blood lipids	67	80	Yes ^‡^
(f) Diabetes	100	80	Yes
(g) High blood pressure	100	90	Yes
(h) Migraine	83	80	Yes
(i) Valvular heart disease	67	67	No
(j) Prolonged bed rest, including planned surgery	67	80	No
*If applicable:* Have you already discussed this situation with a physician (regarding your HC)?	100	88	Yes

Participants: *n* = 17; **^◊^** = Consensus defined a priori based on 70% agreement in both groups; **^&^** = Inclusion backed-up with a tie-breaker question; **^‡^** = Inclusion due to feedback/voting from the first cycle.

## Supplementary Materials

The final supporting material (in German) and a summary of the final protocol in English can be downloaded at: https://www.mdpi.com/article/10.3390/pharmacy10060168/s1.
