# Supplementary material for: Development of a Protocol Using the Delphi Method for the ad interim Supply of Hormonal Contraceptives in Swiss Pharmacies
Source: Pharmacy (Basel). 2022 Dec 2;10(6):168. doi: 10.3390/pharmacy10060168 (PMC9785210; doi:10.3390/pharmacy10060168)
Supplement: Supplementary file 1 [file pharmacy-10-00168-s001.zip › 221121 Supplement_Protocol_FINAL_tyo.pdf]

Protocol for Pharmacies – for Overview Purposes only: The original material resulting from our Delphi survey is available in German. This protocol aiming to verify and document eligibility to HC in women who are already using it but have no or no valid prescription (*ad interim* supply of HC in pharmacies according to the current Swiss law (HMG/TPA)) was translated for overview purposes only.

**Clarification/Documentation**	**Answer Choice(s)**
Name and surname / Date of birth or age	____________ / ____________
**I) Short Clarification**	
1) What is the reason for the urgent supply of HC without Rx?	☐Rx expired on...<br>☐Rx not available<br>☐HC are usually obtained from the physician<br>☐Foreign Rx<br>☐Other: ________
2a) Have HC been prescribed by a physician within the last 2 years?	☐Yes→ see 2b) / ☐No#
2b) Which product do you take, or do you use?	Product name or active ingredient(s): ________<br>☐CHC / ☐Progesterone-only
2c) When did you last take, or use the product?	Date (time, if applicable):________
2d) Do you tolerate the product well?	☐Yes / ☐No# ________
3) When was the last gynecological check-up?	Month/Year: ________
4a) When did the last menstrual bleeding start?	Date: ________
4b) Was the last menstrual bleeding as usual?	☐Yes / ☐No#
**Initial Evaluation by the Pharmacist**	
5) Can a pregnancy be excluded with a high degree of probability?	☐Yes / ☐No#
6) Is the detailed clarification advisable?	☐Yes→ see II) / ☐No→ see III) & IV)
Notes:	
**II) Detailed Clarification**	
7a) Does one or more of the following situations apply to you? *(tick applicable situation(s) and continue with 7b, respectively with 8a)*	☐Age > 35 year<br>☐Smoking<br>☐High blood pressure<br>☐Overweight<br>☐High blood lipids<br>☐Diabetes<br>☐History of blood clots, including 1st degree relative<br>☐Tendency to thrombosis/Thrombophilia<br>☐Migraine<br>☐Childbirth < 6 weeks<br>☐Breastfeeding<br>☐Vaginal bleeding not clarified<br><br>☐No → *see 8a)*
7b) Have you already discussed this situation with a physician (regarding HC)?	☐Yes / ☐No#
8a) What other drugs are you taking or using?	________ → *see 8b)* / ☐None → *see III)*
8b) Drug evaluation by the pharmacist	CYP3A4-inducing drugs: ☐Yes / ☐No<br>Relevant CI or IA: ☐Yes / ☐No
Notes:	

Abbreviations: CI = Contraindication(s); HC = Hormonal contraceptive(s); HMG/TPA = "Heilmittelgesetz"/Therapeutic Products Act; IA = Interaction(s); CHC = Combined hormonal contraceptive(s); Rx = Prescription; SGGG = Swiss Society for Gynecology and Obstetrics; SS = Pregnancy

III) Information Given to the Woman

☐ Recommendation for regular gynecological check-ups if the last gynecological check-up was more than 1 year ago (re-evaluation of contraception, breast examination, cancer smear, etc.)

☐ General information on the female cycle

☐ Information on the mechanism of action of HC

☐ Product information (e.g., instructions; when its effectiveness may be limited, measures in case of forgotten pill, etc.)

☐ Information on different contraceptive methods (see e.g., factsheet www.sex-i.ch)

☐ Information on sexually transmitted infections/diseases (see e.g., www.lovelife.ch)

☐ Information on the detection of thromboses/embolisms (see e.g., information sheet for users of the SGGG)

☐ Other: ____________

IV) Documentation of the Dispensing Decision

Capability of judgment is given	☐ Yes / ☐ No
Risk or CI present	☐ Yes / ☐ No
Supply of HC	☐ Yes* / ☐ No
Referral	☐ Yes§ ☐ No
Notes:	
Date & Signature of pharmacist	

#=where appropriate: referral to a physician

*=Placeholder for product name and pack size

§=Placeholder for referral information, e.g., physician's name/address

Abbreviations: CI = Contraindication(s); HC = Hormonal contraceptive(s); HMG/TPA = "Heilmittelgesetz"/Therapeutic Products Act; IA = Interaction(s); CHC = Combined hormonal contraceptive(s); Rx = Prescription; SGGG = Swiss Society for Gynecology and Obstetrics; SS = Pregnancy
