# Supplementary material for: Development of a Protocol Using the Delphi Method for the ad interim Supply of Hormonal Contraceptives in Swiss Pharmacies
Source: Pharmacy (Basel). 2022 Dec 2;10(6):168. doi: 10.3390/pharmacy10060168 (PMC9785210; doi:10.3390/pharmacy10060168)
Supplement: Supplementary file 1 [file pharmacy-10-00168-s001.zip › Supplement_Abgabeprotokoll__German.pdf]

Abgabeprotokoll hormonelle Kontrazeptiva ohne ärztliche Verordnung

(im begründeten Ausnahmefall gemäss HMG)

Name & Vorname der Kundin	
Geburtsdatum bzw. Alter	

I) Kurzabklärung

1. Was ist der Grund für die Abgabe von HK ohne ärztliche Verordnung (Rezept)?	☐ Rezept abgelaufen am: ______<br>☐ Rezept nicht dabei<br>☐ HK werden sonst beim Arzt bezogen<br>☐ Ausländisches Rezept<br>☐ Andere: ______
2. a) Wurden HK innerhalb der letzten 2 Jahre von einem Arzt/einer Ärztin verordnet?	☐ Ja → weiter bei Frage 2b ☐ Nein → ggf. Weiterleitung Arzt/Ärztin
b) Welches Präparat nehmen Sie ein bzw. wenden Sie an?	Präparatename bzw. Wirkstoff(e): ______<br>☐ KHK ☐ Gestagenmonopräparat
c) Wann haben Sie das Präparat zum letzten Mal eingenommen bzw. angewendet?	Datum (evt. Uhrzeit): ______
d) Vertragen Sie das Präparat gut?	☐ Ja ☐ Nein ______ → ggf. Weiterleitung Arzt/Ärztin
3. Wann hat die letzte gynäkologische Routinekontrolle stattgefunden?	Monat/Jahr: ______
4. a) Wann hat die letzte Periode begonnen?	Datum: ______
b) Verlief die letzte Periode wie üblich?	☐ Ja ☐ Nein → ggf. Weiterleitung Arzt/Ärztin

Erste Evaluation der Situation durch den Apotheker/die Apothekerin:

5. Kann eine SS mit grosser Wahrscheinlichkeit ausgeschlossen werden?	☐ Ja ☐ Nein → ggf. Weiterleitung Arzt/Ärztin
6. Ist eine ausführlichere Abklärung sinnvoll?	☐ Ja → weiter mit Abschnitt II ☐ Nein → weiter mit Abschnitt III & IV

Notizen:

Abkürzungen: *HK = hormonelle Kontrazeptiva; HMG = Heilmittelgesetz; IA = Interaktion; KHK = kombinierte hormonelle Kontrazeptiva; KI = Kontraindikation; SS = Schwangerschaft*

II) Ausführlichere Abklärung (im Bedarfsfall zusätzlich auszufüllen)

7. a) Treffen bei Ihnen eine oder mehrere der folgenden Situationen zu?	☐ Alter > 35 Jahre<br>☐ Rauchen<br>☐ Bluthochdruck<br>☐ Übergewicht<br>☐ Erhöhte Blutfettwerte<br>☐ Diabetes («Zuckerkrankheit»)<br>☐ Auftreten von einem Blutgerinnsel bei Ihnen oder einem Verwandten 1. Grades<br>☐ Thromboseneigung/ Thrombophilie<br>☐ Migräne<br>☐ Geburt < 6 Wochen<br>☐ Stillzeit<br>☐ Nicht abgeklärte vaginale Blutungen<br>zutreffende Situation(en) ankreuzen<br>→ weiter bei Frage 7b
	☐ Nein → Frage 8a
b) Wurde die Situation im Zusammenhang mit Ihrer Verhütungsmethode bereits mit einem Arzt/einer Ärztin besprochen?	☐ Ja ☐ Nein<br>→ ggf. Weiterleitung Arzt/Ärztin
8. a) Welche weiteren Arzneimittel nehmen Sie ein bzw. wenden Sie an?	☐ ________________________________<br>→ weiter bei Frage 8b<br>☐ Keine<br>→ weiter mit Abschnitt III
b) Evaluation der Arzneimittel durch Apotheker:in	CYP3A4-induzierende Arzneimittel: ☐ Ja ☐ Nein<br>Relevante KI bzw. IA: ☐ Ja ☐ Nein

Notizen:

Abkürzungen: *HK* = *hormonelle Kontrazeptiva; HMG = Heilmittelgesetz; IA = Interaktion; KHK = kombinierte hormonelle Kontrazeptiva; KI = Kontraindikation; SS = Schwangerschaft*

III) Welche Informationen wurden der Kundin während des Gesprächs gegeben?

- ☐ Empfehlung für regelmässige gynäkologische Kontrollen, falls die letzte gyn. Untersuchung bereits mehr als 1 Jahr zurück liegt (Reevaluation der Verhütung, Brustuntersuchung, Krebsabstrich etc.)
- ☐ Informationen allgemein zum weiblichen Zyklus
- ☐ Informationen zum Wirkmechanismus von HK
- ☐ Produkteinformationen (z. B. Einnahmehinweise; wann kann die Wirksamkeit eingeschränkt sein, Massnahmen bei vergessener Pille etc.)
- ☐ Informationen über verschiedene Verhütungsmethoden (siehe z. B. Factsheet www.sex-i.ch)
- ☐ Informationen zu sexuell übertragbaren Erkrankungen (siehe z. B. www.lovelife.ch)
- ☐ Informationen zur Erkennung von Thrombosen/Embolien (siehe z. B. Informationsblatt der SGGG für Anwenderinnen von KHK)
- ☐ Weitere:

IV) Dokumentation des Abgabeentscheids

Urteilsfähigkeit gegeben	☐ Ja	☐ Nein
Risiko / KI vorhanden?	☐ Ja	☐ Nein
Abgabe von HK	☐ Ja; evt. Produkt & Packungsgrösse: ________________________________	☐ Nein
Weiterleitung der Kundin	☐ Ja; an: ________________________________	☐ Nein
Notizen		
Datum & Visum Apotheker:in		

***Abkürzungen**: **HK** = hormonelle Kontrazeptiva; **HMG** = Heilmittelgesetz; **IA** = Interaktion; **KHK** = kombinierte hormonelle Kontrazeptiva; **KI** = Kontraindikation; **SS** = Schwangerschaft*
