# Supplementary material for: Development of a Protocol Using the Delphi Method for the ad interim Supply of Hormonal Contraceptives in Swiss Pharmacies
Source: Pharmacy (Basel). 2022 Dec 2;10(6):168. doi: 10.3390/pharmacy10060168 (PMC9785210; doi:10.3390/pharmacy10060168)
Supplement: Supplementary file 1 [file pharmacy-10-00168-s001.zip › Supplement_Kommentarblatt_German.pdf]

<u>Kommentare:</u> Abgabeprotokoll hormonelle Kontrazeptiva ohne ärztliche Verordnung (im begründeten Ausnahmefall gemäss HMG)

I) Kurzabklärung

1.	Was ist der Grund für die Abgabe von HK ohne ärztliche Verordnung?	• Evaluation und Dokumentation der Situation (Ausnahmefall)
2.	a) Wurden HK innerhalb der letzten 2 Jahre von einem Arzt/einer Ärztin verordnet?	• Empfehlung für regelmässige gyn. Kontrollen aussprechen, falls letzte Konsultation > 1 Jahr<br>• Abklärung bezüglich relevanter Kontraindikationen und Risikofaktoren (siehe Abschnitt II)<br>• je nach Situation → Weiterleitung an einen Arzt/eine Ärztin
	b) Welches Präparat nehmen Sie ein bzw. wenden Sie an?	• unterschiedliche Eigenschaften und KI beachten (KHK mit erhöhtem Risikopotenzial)
	c) Wann haben Sie das Präparat zum letzten Mal eingenommen bzw. angewendet?	• Einnahmeintervall korrekt oder evt. bereits verlängert?<br>o Besteht das Risiko für eine SS, z. B. durch Verhütungspanne?<br>o ggf. spezifische Massnahmen empfehlen: vgl. «Differenziertes Vorgehen bei fehlerhafter Anwendung einer hormonellen Verhütung»[1]<br>• evt. erfragen, wann die nächste Einnahme/Anwendung erfolgt → Dringlichkeit der Abgabe ohne ärztliche Verordnung abschätzen<br>• bei «Restart» (=erneute Einnahme von HK nach einem Unterbruch):<br>o Grund und Dauer für den Unterbruch? Mit Arzt/Ärztin besprochen?<br>o Cave: Thromboserisiko v. a. im 1. Anwendungsjahr erhöht: ausführliche Evaluation notwendig → Weiterleitung an Arzt/Ärztin empfohlen
	d) Vertragen Sie das Präparat gut?	• Gab es mit der bisherigen Therapie Probleme bezüglich Verträglichkeit oder bei der Anwendung? → ggf. Weiterleitung an Arzt/Ärztin
3.	Wann hat die letzte gynäkologische Routinekontrolle stattgefunden?	• Reevaluation der Verhütung bzw. gyn. Kontrolle in der Regel alle 1 bis 2 Jahre<br>• Kundin ggf. über die Wichtigkeit und den Nutzen von regelmässigen gynäkologischen Untersuchungen informieren (insbesondere, wenn die letzte Untersuchung bereits > 1 Jahr her ist)
4.	a) Wann hat die letzte Periode begonnen?	• Orientierung im Zyklus zur Abschätzung der Dringlichkeit der Abgabe
	b) Verlief die letzte Periode wie üblich?	• Hinweis zum Ausschluss einer möglichen SS<br>• bei Veränderungen (z. B. ungewohnt kürzere/schwächere Periode) → ggf. Weiterleitung Arzt/Ärztin
**Evaluation der Situation durch den Apotheker/die Apothekerin:**		
5.	Kann eine SS mit grosser Wahrscheinlichkeit ausgeschlossen werden?	• Ausschluss einer SS: z. B. kein GV seit der letzten Periode od. korrekte und konsequente Anwendung einer verlässlichen Verhütungsmethode<br>• falls eine SS <u>nicht</u> ausgeschlossen werden kann → SS-Test durchführen (frühestens 21 Tage nach dem letzten uGV aussagekräftig) bzw. Weiterleitung an Arzt/Ärztin
6.	Ist eine ausführlichere Abklärung sinnvoll?	• Insbesondere bei fehlendem Nachweis für bisherige Verordnung von HK oder bei Unsicherheiten bezüglich der Eignung für HK

Abkürzungen: *GV = Geschlechtsverkehr; HK = hormonelle Kontrazeptiva; HMG = Heilmittelgesetz; IA = Interaktion; KHK = kombinierte hormonelle Kontrazeptiva; KI = Kontraindikation; SS = Schwangerschaft; uGV = ungeschützter Geschlechtsverkehr; UKMEC = Medical Eligibility Criteria for Contraceptive Use*

II) Ausführlichere Abklärung (im Bedarfsfall zusätzlich ausfüllen)

<table>
<tr>
<td>7.</td>
<td>a) Treffen bei Ihnen eine oder mehrere der folgenden Situationen zu?</td>
<td>
Erhöhtes Risiko bzw. mögliche Kontraindikation in folgenden Situationen (insbesondere für KHK):

Alter > 35 Jahre: erhöhtes Thromboserisiko unter KHK; insbesondere für Raucherinnen
Rauchen: erhöhtes Thromboserisiko unter KHK (altersabhängig)
ggf. Rauchstopp-Beratung anbieten
Bluthochdruck: Risikofaktor für thromboembolische Ereignisse
Übergewicht: erhöhtes Thromboserisiko, deshalb mögliche KI für KHK
erhöhte Blutfettwerte: stark erhöhte Blutfettwerte als KI für KHK
Diabetes («Zuckerkrankheit»): Endorganschädigungen als KI für KHK
Blutgerinnsel/Thromboseneigung: z. B. Hirn-, Herzinfarkt, Beinvenenthrombose, Lungenembolie, bekannte Thrombophilie
Verwandte 1. Grades = Eltern und ihre Kinder
ggf. an die typischen Symptome erinnern; in diesen Fällen sollte die Frau sofort einen Arzt/eine Ärztin kontaktieren
Migräne: Risikofaktor für Schlaganfälle
insbesondere Migräne mit Aura als KI für KHK
Geburt < 6 Wochen: erhöhtes Thromboserisiko in den ersten Wochen nach der Geburt
KI für KHK für mind. 6 Wochen
«Restart» von HK nach der Geburt durch den Arzt/die Ärztin
Stillzeit: häufig werden Gestagenmonopräparate verordnet

Für weitere Situationen oder Informationen siehe z. B. UKMEC[2].
</td>
</tr>
<tr>
<td></td>
<td>b) Wurde die Situation im Zusammenhang mit Ihrer Verhütungsmethode bereits mit einem Arzt/einer Ärztin besprochen?</td>
<td>

«Ja» → regelmässige Reevaluation durch Arzt/Ärztin sinnvoll
«Nein» → Weiterleitung an Arzt/Ärztin zur genauen Evaluation

</td>
</tr>
<tr>
<td>8.</td>
<td>a) Welche weiteren Arzneimittel nehmen Sie ein bzw. wenden Sie an?

b) Evaluation der Arzneimittel durch Apotheker:in</td>
<td>

Arzneimittel auf potenzielle KI bzw. IA überprüfen
z. B. CYP3A4-Induktor(en), die zu einer verminderten Wirksamkeit von gewissen HK führen können (z. B. Carbamazepin, Phenytoin, Rifampicin)
auch Arzneimittel berücksichtigen, die durch HK beeinflusst werden können (z. B. Lamotrigin, Valproat)
falls relevante KI/IA vorhanden:
Sind Kundin und Arzt/Ärztin informiert und werden entsprechende Massnahmen getroffen? (z. B. zusätzliche Verhütung mit Kondom)
ggf. weitere Abklärungen treffen bzw. Weiterleitung an Arzt/Ärztin

</td>
</tr>
</table>

__Abkürzungen__: __GV__ = Geschlechtsverkehr; __HK__ = hormonelle Kontrazeptiva; __HMG__ = Heilmittelgesetz; __IA__ = Interaktion; __KHK__ = kombinierte hormonelle Kontrazeptiva; __KI__ = Kontraindikation; __SS__ = Schwangerschaft; __uGV__ = ungeschützter Geschlechtsverkehr; __UKMEC__ = Medical Eligibility Criteria for Contraceptive Use

III) Welche Informationen wurden der Kundin während des Gesprächs gegeben?

Nützliche Links:

- Informationen über verschiedene Verhütungsmethoden (siehe z. B. Factsheet www.sex-i.ch)
- Informationen zu sexuell übertragbaren Erkrankungen (siehe z. B. www.lovelife.ch)
- Informationen zur Erkennung von Thrombosen/Embolien (siehe z. B. Informationsblatt der SGGG für Anwenderinnen von KHK)

IV) Dokumentation des Abgabeentscheids

Urteilsfähigkeit	• Urteilsfähig im Sinne des Gesetzes ist jede Person, der nicht wegen ihres Kindesalters, infolge geistiger Behinderung, psychischer Störung, Rausch oder ähnlicher Zustände die Fähigkeit mangelt, vernunftgemäss zu handeln. (gemäss Schweizerisches Zivilgesetzbuch, Art. 16A)<br>• ≥ 16 Jahre: Urteilsfähigkeit wird angenommen<br>• < 16 Jahre: Urteilsfähigkeit muss durch Apotheker:in beurteilt und dokumentiert werden
Abgabeentscheid & Weiterleitung der Kundin	• Die Risikosituation soll gegenüber dem Risiko für eine ungewollte Schwangerschaft abgewogen werden.<br>• Der Abgabeentscheid erfolgt situativ und liegt im Ermessen des verantwortlichen Apothekers bzw. der verantwortlichen Apothekerin.

Weitere Informationen für Fachpersonen:

1	Differenziertes Vorgehen bei fehlerhafter Anwendung einer hormonellen Verhütung<br>Quelle: www.sexuelle-gesundheit.ch
2	UKMEC Summary Table 2019 (UK Medical Eligibility Criteria for Contraceptive Use)<br>Quelle: www.fsrh.org

Referenzen (alphabetisch):

- Checkliste CHC für Erst- oder Neuverschreibungen (Expertenbrief Nr. 72) – www.sggg.ch (Stand 4/2021)
- FSRH Guideline Quick Starting Contraception – www.fsrh.org (Stand 2017)
- Informationsblatt für Anwenderinnen kombinierter hormonaler Kontrazeptiva – www.sggg.ch (Stand 4/2021)
- Speroff's Clinical Gynecologic Endocrinology and Infertility (9th Edition; 2019)
- Summary Chart of U.S. Medical Eligibility Criteria for Contraceptive Use – www.cdc.gov (Stand 2020)
- UKMEC Summary Table – UK Medical Eligibility Criteria for Contraceptive Use – www.fsrh.org (Stand 2019)

__Abkürzungen__: __GV__ = Geschlechtsverkehr; __HK__ = hormonelle Kontrazeptiva; __HMG__ = Heilmittelgesetz; __IA__ = Interaktion; __KHK__ = kombinierte hormonelle Kontrazeptiva; __KI__ = Kontraindikation; __SS__ = Schwangerschaft; __uGV__ = ungeschützter Geschlechtsverkehr; __UKMEC__ = Medical Eligibility Criteria for Contraceptive Use
